# Characterization of a Topramezone-Resistant Rice Mutant TZR1: Insights into GST-Mediated Detoxification and Antioxidant Responses

**DOI:** 10.3390/plants14030425

**Published:** 2025-02-01

**Authors:** Shiyuan Hu, Kai Luo, Tao Tang, Guolan Ma, Yajun Peng, Yuzhu Zhang, Yang Liu, Lang Pan, Sifu Li

**Affiliations:** 1 College of Plant Protection, Hunan Agricultural University, Changsha 410128, China; hushiyuan@stu.hunau.edu.cn (S.H.); luokai@stu.hunau.edu.cn (K.L.); 2 Institute of Plant Protection, Hunan Academy of Agricultural Sciences, Changsha 410125, China; tangtao@hunaas.cn (T.T.); maguolan@hunaas.cn (G.M.); pengyajun@hunaas.cn (Y.P.); 3 State Key Laboratory of Hybrid Rice, Hunan Hybrid Rice Research Center, Changsha 410125, China; zhangyuzhu@hunaas.cn (Y.Z.); liuyang@hhrrc.ac.cn (Y.L.)

**Keywords:** radiation mutagenesis, topramezone, HPPD, glutathione *S*-transferase, UDP-glycosyltransferase, ATP-binding cassette transporter

## Abstract

Mutagenesis breeding, combined with the application of corresponding herbicides to develop herbicide-resistant rice germplasm, provides great promise for the management of weeds and weedy rice. In this study, a topramezone-resistant rice mutant, TZR1, was developed from the indica rice line Chuangyu 9H (CY9H) through radiation mutagenesis and topramezone selection. Dose–response curves revealed that the resistance index of TZR1 to topramezone was 1.94-fold compared to that of CY9H. The resistance mechanism of TZR1 was not due to target-site resistance. This resistance could be reversed by a specific inhibitor of glutathione *S*-transferase (GST). The activity of antioxidant enzymes was analyzed. SNPs and Indels were detected using whole-genome resequencing; differentially expressed genes were identified through RNA sequencing. Then, they underwent Gene Ontology and Kyoto Encyclopedia of Genes and Genomes enrichment analyses. Key candidate genes associated with topramezone resistance were validated via a real-time quantitative PCR assay. Five GST genes, two UDP-glycosyltransferase genes, and three ATP-binding cassette transporter genes were identified as potential contributors to topramezone detoxification in TZR1. Overall, these findings suggest that GST enzymes possibly play an important role in TZR1 resistance to topramezone. This study will provide valuable information for the scientific application of 4-hydroxyphenylpyruvate dioxygenase inhibitors in paddy fields in future.

## 1. Introduction

Rice (*Oryza sativa* L.), one of the most important staple crops worldwide, plays a crucial role in ensuring food security and feeds nearly half of the world’s population [1]. In Asia, rice cultivation predominantly involves two methods: direct seeding and transplanting [2]. Among these, direct seeding has become more and more popular due to its labor-saving and cost-reducing advantages in rice production. However, this technique faces severe challenges from weeds and weedy rice (*Oryza sativa* f. spontanea), which compete with cultivated rice for sunlight, nutrients, water, and growing space, leading to substantial yield losses and quality degradation [3,4]. Currently, one of the most effective strategies for weed control in the direct seeding of rice is the use of chemical herbicides. However, the continuous and frequent use, or even the abuse of herbicides, has caused the development of herbicide-resistant weed species [5]. Additionally, due to the genetic similarity between weedy rice and cultivated rice, most herbicides with high safety for the latter are ineffective against the former [6]. Hence, developing herbicide-resistant rice varieties is of great significance for effectively managing both weeds and weedy rice in rice production.

**Figure 1:**
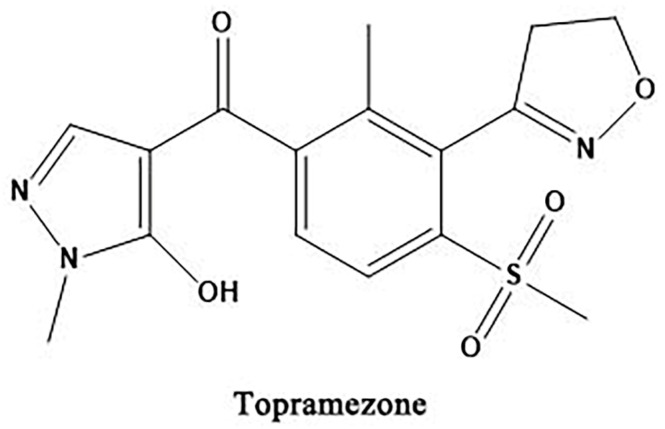
The chemical structure of topramezone.

Topramezone is a 4-hydroxyphenylpyruvate dioxygenase (HPPD) inhibitor developed by BASF SE (Ludwigshafen, Germany). It is classified as a benzoylpyrazolone herbicide, with an excellent safety profile, high selectivity, broad-spectrum herbicidal activity, long-term efficacy, and better environmental compatibility for use in cornfields (Figure 1) [7]. Rice exhibits high sensitivity to topramezone. Therefore, if its application can be extended to paddy fields, it will serve as an effective tool for controlling herbicide-resistant weeds and weedy rice.

Herbicide resistance mechanisms are generally categorized into target-site resistance (TSR) and non-target-site resistance (NTSR) mechanisms [8]. TSR primarily involves mutations and/or the overexpression of target enzyme genes [9]. To date, no reports have documented mutations of the HPPD gene conferring weed resistance to HPPD inhibitors. However, many HPPD genes with resistance to HPPD inhibitors have been cloned in plants such as the mesotrione-resistant HPPD gene from oat (*Avena sativa* L.) and the topramezone-resistant HPPD gene from Japanese goldthread (*Coptis japonica* Makino) [10,11]. Additionally, some HPPD genes resistant to HPPD inhibitors have been successfully obtained by site-directed and saturation mutagenesis. For instance, the double mutations of Glu-258-Met and Try-333-Phe, as well as the single mutation of Asn-321-Tyr or Glu-322-Val in the *Sphingobium* sp. HPPD gene, enhanced resistance to topramezone [12,13]. In contrast, NTSR primarily involves mechanisms such as reduced herbicide absorption and translocation, vacuolar sequestration, and enhanced herbicide metabolism [14,15]. Several plant detoxification proteins have been potentially associated with NTSR. Among these, only four proteins have been identified as being involved in NTSR, as follows: cytochrome P450 monooxygenases (CYP450s); glutathione *S*-transferases (GSTs); UDP-glycosyltransferases (UGTs); and ATP-binding cassette (ABC) transporters. Regarding NTSR, the detoxification of herbicides primarily comprises four stages. In phase I, activation occurs as CYP450s or mixed-function oxidases expose functional groups to facilitate subsequent enzymatic binding. Phase II involves conjugation, where large hydrophilic molecules, such as sugars or sulfhydryl groups, bind to the activated herbicide molecules; this process is mainly executed by GSTs or UGTs. Phase III involves transport, which is typically facilitated by ABC transporters that actively shuttle the conjugated herbicide molecules to the vacuole or extracellular space. During phase IV, further degradation of the herbicide molecules occurs within the vacuole or extracellular space [16].

To date, reports on resistance mechanisms to HPPD inhibitors are limited and have only been observed in both grass and broadleaf weeds. For example, Chinese sprangletop (*Leptochloa chinensis* L.) is resistant to tripyrasulfone [17]; green foxtail [*Setaria viridis* (L.) P. Beauv.], redroot pigweed (*Amaranthus retroflexus* L.), palmer amaranth (*A. palmeri* S. Watson) and tall waterhemp [*A. Tuberculatus* (Moq.) JD Sauer] are resistant to mesotrione [18,19,20,21], and wild radish (*Raphanus raphanistrum* L.) is resistant to pyrasulfotole and topramezone [22].

The combined use of herbicides with corresponding herbicide-resistant rice varieties has proven to be an effective strategy for controlling resistant weeds and weedy rice in paddy fields [23]. In recent years, increasing attention has been paid by researchers to the development of herbicide-resistant rice through genetic breeding approaches [24]. Mutagenesis breeding is one of the most common methods used in genetic breeding and includes chemical mutagenesis and radiation mutagenesis. Chemical mutagenesis is typically used with ethyl methanesulfonate (EMS) to treat rice seeds, while radiation mutagenesis employs X-rays, γ-rays, and heavy ion beams to treat rice seeds [23]. For instance, a glufosinate-resistant mutant (*gar6-2*) from the rice line Longgeng 31 was produced by EMS mutagenesis, while two glufosinate-resistant mutants (*glr1* and *glr2*) from Jingeng 818 were obtained with heavy ion beam irradiation [1,25].

In this study, a topramezone-resistant rice mutant (TZR1) was first developed through the heavy ion beam irradiation of seeds from the indica rice line Chuangyu 9H (CY9H), and was further selected by topramezone. The first objective of this study was to evaluate the resistance level of TZR1 to topramezone using the whole plant method. Second, the *OsHPPD* gene was cloned with a PCR assay and the full-length cDNA of the *OsHPPD* gene was analyzed by SnapGene 7.0.2 software (GSL Biotech LLC, Boston, MA, USA). Its relative expression levels were determined using a real-time quantitative PCR (RT-qPCR) assay. Third, the impacts of CYP450 and GST inhibitors on the resistance of TZR1 to topramezone were assessed, along with measurements of antioxidant enzyme activity. Additionally, SNPs and Indels were identified by whole-genome resequencing (WGS). Differentially expressed genes (DEGs) were detected by transcriptome sequencing (RNA-seq). Then, key candidate genes associated with topramezone resistance were validated using an RT-qPCR assay. The present study will contribute towards clarifying the resistance mechanism of TZR1 to topramezone and the development of topramezone-resistant rice germplasm. Our study provides a theoretical foundation for the application of topramezone in rice production. In subsequent research, we will further investigate the resistance genes associated with topramezone and elucidate, in depth, their molecular mechanisms in topramezone resistance.

## 2. Results

### 2.1. Dose-Response of CY9H and TZR1 to Topramezone

**Figure 2:**
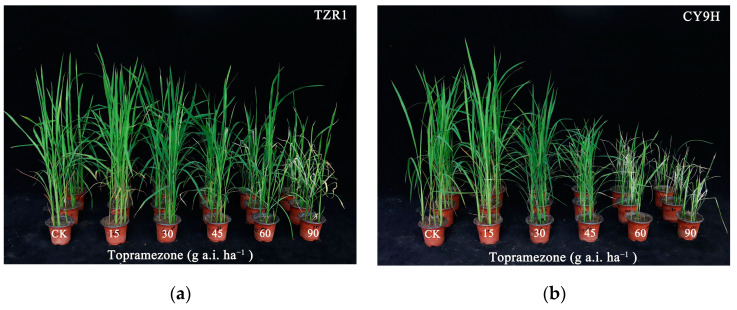
Phenotypic response of plants to topramezone foliar spray at 21 d after treatment: TZR1 (**a**); and CY9H (**b**). Topramezone was applied at 0, 15, 30, 45, 60, and 90 g a.i. ha^−1^ (*n* = 108).

The results indicated a significant difference in the response to topramezone between the TZR1 and CY9H rice lines at 21 d post treatment (Figure 2). The dose of topramezone showed that the growth reduction, by 50% in fresh weight (GR_50_), was 31.27 g a.i. ha^−1^ for CY9H, while 60.51 g a.i. ha^−1^ was observed for TZR1 (Table 1). Both GR_50_ values exceeded the recommended dose of topramezone, ranging from 30 to 36 g a.i. ha^−1^. The resistance index (RI) value was 1.94-fold for TZR1 (Table 1), demonstrating that TZR1 exhibited stronger resistance to topramezone compared to CY9H.

### 2.2. Sequence Analysis and Expression Levels of OsHPPD mRNA

The full-length CDS of the *OsHPPD* was amplified from both CY9H and TZR1 plants. Sequence alignment results revealed that the *OsHPPDs* of CY9H and TZR1 shared a 100% identity with the reference gene of *Os02g0168100*; in other words no base deletions or mutations were detected. These results indicate that TZR1 resistance to topramezone is not due to mutations in the *OsHPPD* gene.

**Figure 3:**
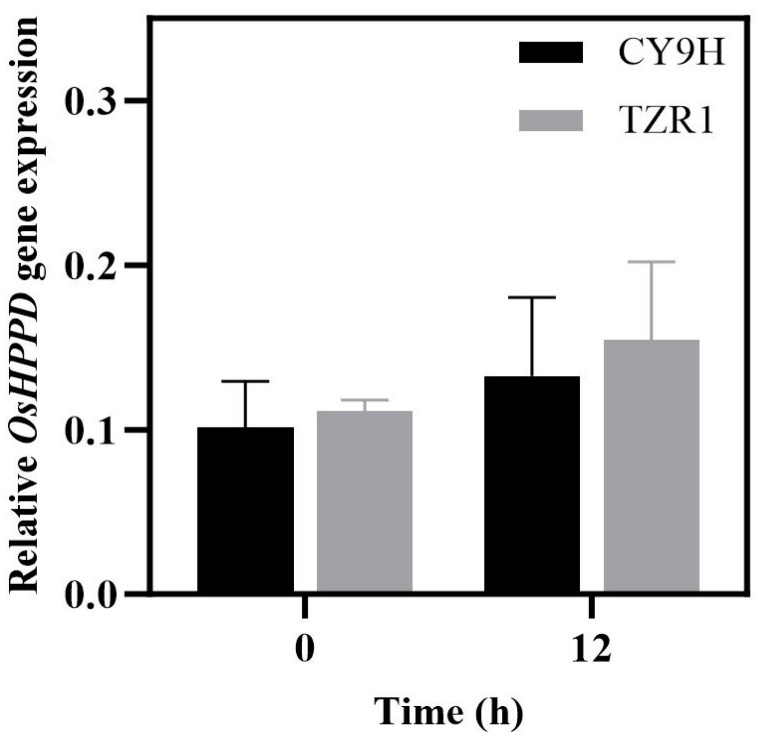
Relative expression levels of OsHPPD mRNA in CY9H and TZR1 at 0 and 12 h after topramezone treatment (FC < 2, *p* > 0.05, *n* = 6). Data are presented as the mean ± standard error (SE).

The results of the RT-qPCR assay showed that the relative expression level of *OsHPPD* mRNA in TZR1 was 1.10-fold before the topramezone treatment (*p* = 0.76, *t*-test), compared with that of CY9H, it was increased to 1.17-fold at 12 h (*p* = 0.75, *t*-test). These results showed that the *OsHPPD* expression levels in CY9H and TZR1 did not vary more than 2-fold, and their differences were not statistically significant (*p* > 0.05; Figure 3). Thus, the *OsHPPD* gene is not responsible for the resistance of TZR1 to topramezone.

### 2.3. Effects of CYP450 and GST Inhibitors on the Resistance of TZR1 to Topramezone

**Figure 4:**
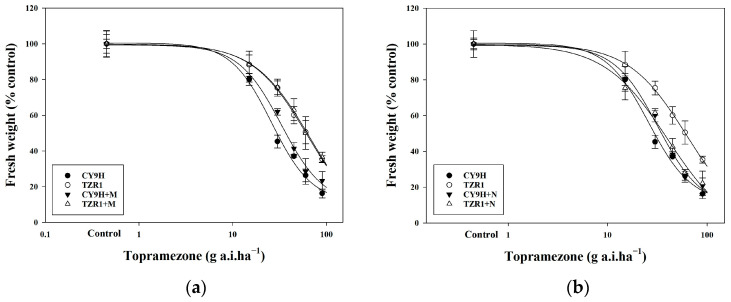
Dose–response curves of CY9H and TZR1 to topramezone. Topramezone was applied at 0, 15, 30, 45, 60, and 90 g a.i. ha^−1^ (*n* = 108): (**a**) response of CY9H and TZR1 to topramezone after treatment with the CYP450 inhibitor malathion (M, 1000 g a.i. ha^−1^); and (**b**) response of CY9H and TZR1 to topramezone after treatment with the GST inhibitor NBD-Cl (N, 270 g a.i. ha^−1^). Data are expressed as mean ± standard error (SE).

Pre-treatment with malathion as the CYP450 inhibitor did not significantly alter the resistance of TZR1 to topramezone with a GR_50_ value of 62.19 g a.i. ha^−1^, which remained similar with that (60.51 g a.i. ha^−1^) of the topramezone treatment alone. In contrast, pre-treatment with the GST inhibitor 4-chloro-7-nitrobenzoxadiazole (NBD-Cl) significantly enhanced the toxicity of topramezone to TZR1, with a GR_50_ value of 36.00 g a.i. ha^−1^ (Table 1). These results indicate that malathion did not change TZR1 resistance to topramezone, whereas NBD-Cl effectively changed this resistance (Figure 4), suggesting that GST may play an important role in TZR1 resistance to topramezone.

### 2.4. Whole-Genome Resequencing Analysis

The WGS results indicated that the clean reads for the TZR1 and CY9H samples were at least 37,420,942 and 33,259,450, with corresponding clean bases of at least 5,605,967,128 bp and 4,980,395,520 bp, respectively. A total of 35.48 Gb of clean data was generated; the Q30 base percentage was above 95.32%. The clean reads of each sample were aligned with the reference genome, yielding alignment efficiencies between 97.62% and 99.31%, with an average coverage depth of at least 12.31× and a genome coverage ranging from 93.99% to 95.68%.

**Figure 5:**
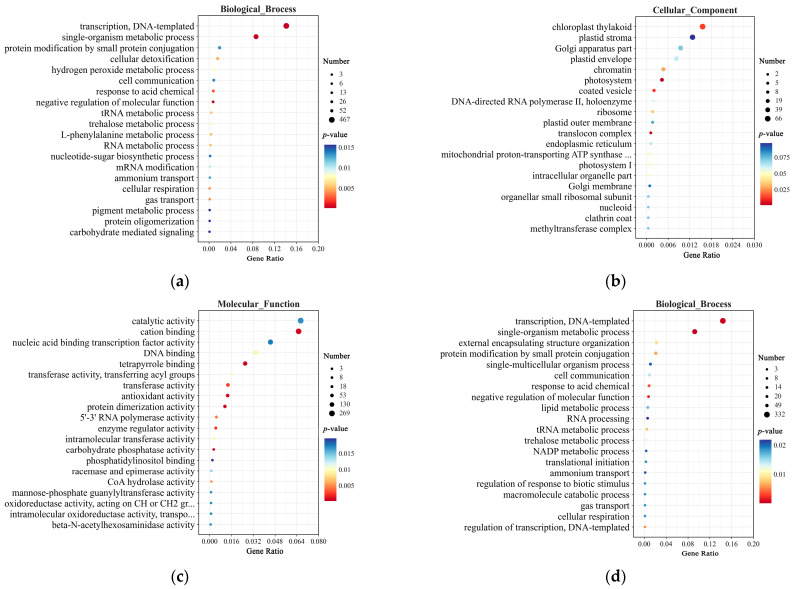

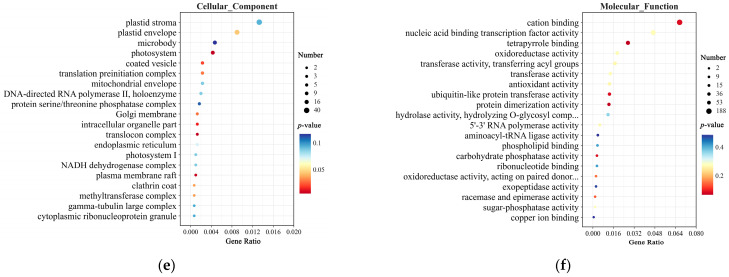
GO enrichment analysis of SNPs and Indels from resequencing data: (**a**) GO enrichment analysis of Biological Process for SNPs; (**b**) GO enrichment analysis of Cellular Component for SNPs; (**c**) GO enrichment analysis of Molecular Function for SNPs; (**d**) GO enrichment analysis of Biological Process for Indels; (**e**) GO enrichment analysis of Cellular Component for Indels; and (**f**) GO enrichment analysis of Molecular Function for Indels. The enrichment analysis yielded the top 20 pathways ranked by the lowest *p*-values, ordered in ascending Gene Ratio.

Gene Ontology (GO) enrichment analysis of the SNPs identified between TZR1 and CY9H revealed 5918 mutated genes annotated in the Biological Process, Cellular Component, and Molecular Function categories (Figure 5). In the Biological Process, the differential genes (DGs) were enriched in pathways such as the single-organism metabolic process, cellular detoxification, and hydrogen peroxide metabolic process (Figure 5a). In the Cellular Component, the DGs were primarily enriched in the chloroplast thylakoid, plastid stroma, and photosystem pathways (Figure 5b). In Molecular Function, the DGs were enriched in catalytic activity, transferase activity, and antioxidant activity (Figure 5c). Among the Indels detected between TZR1 and CY9H, 4154 mutated genes were annotated in the same three categories. In the Biological Process, these DGs were enriched in pathways such as transcription, DNA-templated, single-organism metabolic process, and negative regulation of molecular function (Figure 5d). In the Cellular Component, they were enriched in pathways including photosystem, plasma membrane raft, and plastid envelope (Figure 5e). In Molecular Function, they were enriched in oxidoreductase activity, transferase activity, and antioxidant activity (Figure 5f).

**Figure 6:**
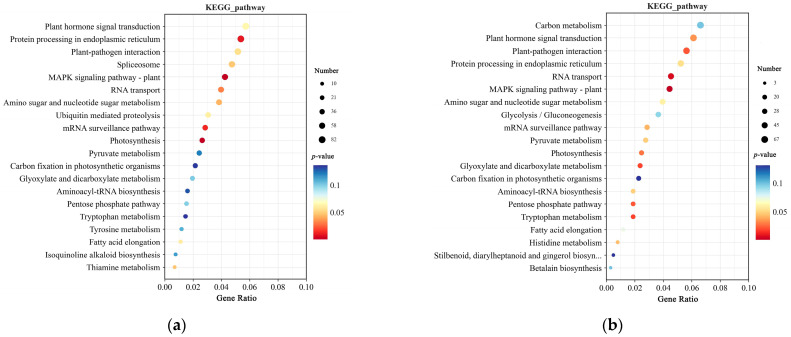
KEGG enrichment analysis of SNPs and Indels from resequencing data: (**a**) KEGG enrichment analysis of SNPs: (**b**) KEGG enrichment analysis of Indels. The enrichment analysis yielded the top 20 pathways ranked by the lowest *p*-values, ordered in ascending Gene Ratio. There is a positive correlation between the size of the bubble and the number of SNPs or Indels. The transition from blue to red via a bubble gradient indicate significant enhancement.

In Kyoto Encyclopedia of Genes and Genomes (KEGG) pathway enrichment, the mutated genes in SNPs were enriched in the MAPK signaling pathway–plant, Photosynthesis, and Protein processing in endoplasmic reticulum (Figure 6a). Mutated genes in Indels were enriched in the MAPK signaling pathway–plant, Photosynthesis, and Plant hormone signal transduction (Figure 6b).

### 2.5. Analysis of DEGs by RNA-Seq

RNA-Seq analysis revealed that, compared to CY9H, there were 2124 DEGs in TZR1 at 0 h, including 1160 upregulated and 964 downregulated DEGs. At 12 h post treatment, there were 2312 DEGs between CY9H and TZR1, containing 1212 upregulated and 1100 downregulated DEGs. Additionally, there were 723 common DEGs at these timepoints.

**Figure 7:**
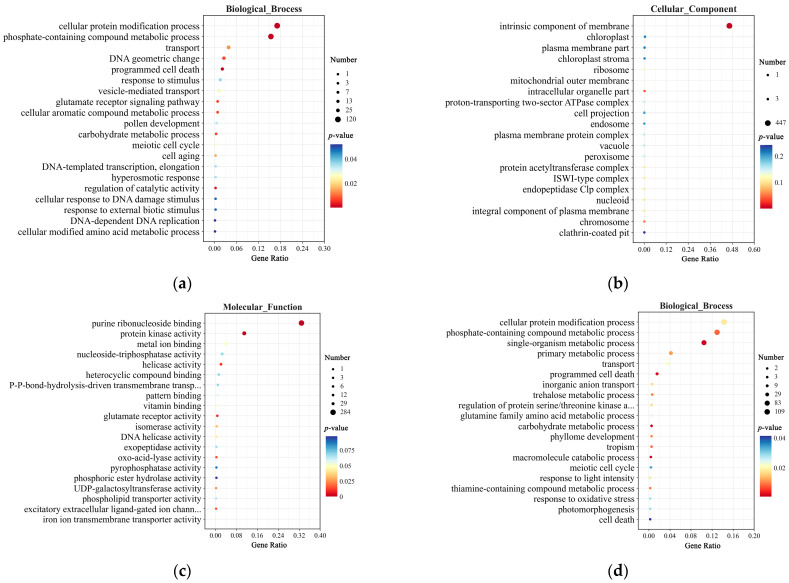

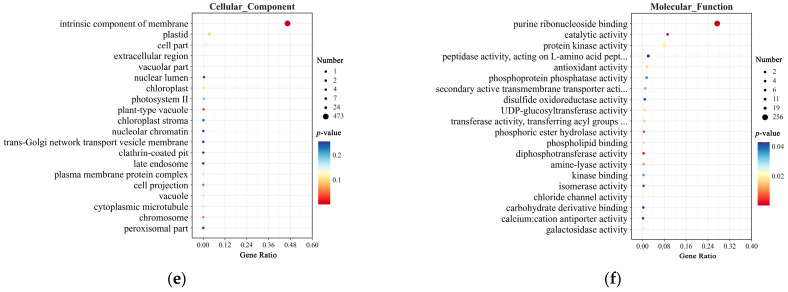
GO enrichment analysis of DEGs in CY9H and TZR1 at 0 and 12 h after topramezone treatment: (**a**) GO enrichment analysis of Biological Process at 0 h; (**b**) GO enrichment analysis of Cellular Component at 0 h; (**c**) GO enrichment analysis of Molecular Function at 0 h; (**d**) GO enrichment analysis of Biological Process at 12 h; (**e**) GO enrichment analysis of Cellular Component at 12 h; and (**f**) GO enrichment analysis of Molecular Function at 12 h. The enrichment analysis yielded the top 20 pathways ranked by the lowest *p*-values, ordered in ascending Gene Ratio.

In the GO enrichment analysis, at 0 h, DEGs in the Biological Process category were enriched in pathways such as cellular protein modification process, transport, response to stimulus, and regulation of catalytic activity (Figure 7a). In the Cellular Component category, they were primarily enriched in the intrinsic component of membrane pathway (Figure 7b). In the Molecular Function category, DEGs were enriched in purine ribonucleoside binding, protein kinase activity, and UDP-galactosyltransferase activity (Figure 7c). At 12 h, DEGs in the Biological Process category were enriched in pathways such as the cellular protein modification process, single-organism metabolic process, transport, carbohydrate metabolic process, and macromolecule catabolic process (Figure 7d). In the Cellular Component category, DEGs were again primarily enriched in the intrinsic component of the membrane pathway (Figure 7e). In the Molecular Function category, DEGs were enriched in pathways such as purine ribonucleoside binding, catalytic activity, antioxidant activity, secondary active transmembrane transporter activity, and UDP-galactosyltransferase activity (Figure 7f).

Interestingly, DEGs at 0 and 12 h were enriched in common pathways, including the cellular protein modification process, transport, carbohydrate metabolic process, intrinsic component of membrane, and purine ribonucleoside binding. Following topramezone treatment, DEGs were also enriched in additional pathways such as macromolecule catabolic process, response to light intensity, response to oxidative stress, catalytic activity, antioxidant activity, and UDP-galactosyltransferase activity. These findings suggest that compared to CY9H, the increased resistance of TZR1 to topramezone may be associated with these enriched pathways.

**Figure 8:**
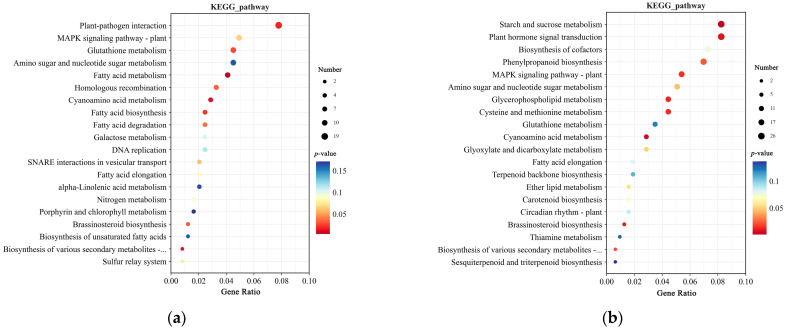
KEGG enrichment analysis of DEGs in CY9H and TZR1 at 0 h and 12 h after topramezone treatment: (**a**) KEGG enrichment analysis at 0 h; and (**b**) KEGG enrichment analysis at 12 h. The enrichment analysis yielded the top 20 pathways ranked by the lowest *p*-values, ordered in ascending Gene Ratio. There is a positive correlation between the size of the bubble and the number of DEGs. The transition from blue to red via a bubble gradient indicates significant enhancement.

KEGG pathway analysis revealed that at 0 h, the top five enriched pathways were Plant-pathogen interaction, MAPK signaling pathway–plant, Glutathione metabolism, Amino sugar and nucleotide sugar metabolism, and Fatty acid metabolism (Figure 8a). At 12 h, the top five enriched pathways were Starch and sucrose metabolism, Plant hormone signal transduction, Biosynthesis of cofactors, Phenylpropanoid biosynthesis, and MAPK signaling pathway–plant (Figure 8b). At 0 and 12 h, DEGs were enriched in the MAPK signaling pathway–plant, Glutathione metabolism, Amino sugar and nucleotide sugar metabolism, Cyanoamino acid metabolism, Brassinosteroid biosynthesis, and Biosynthesis of various secondary metabolites—part 2 pathways. Additionally, at 12 h, DEGs were also enriched in the Carotenoid biosynthesis pathway, which may be associated with TZR1 resistance to topramezone.

### 2.6. Verification of the DEGs by RT-qPCR Assay

According to the RNA-seq results, differential gene expression was filtered using a threshold of *p* < 0.05 and |log2FC| > 1.0. A total of 12 GSTs, 11 UGTs, and 9 ABC transporter genes were found to be upregulated in both TZR1 and CY9H. Further comparison led to the selection of 8 GSTs, 7 UGTs, and 5 ABC transporter genes for RT-qPCR validation. The results revealed that 5 GSTs (*OsGST4*, *OsGSTF1*, *OsGSTU6-1*, *OsGSTU6-3*, *OsGSTT3*), 2 UGTs (*OsUGT73D1* and *OsUGT75L6*), and 3 ABC transporters (*OsABCC8*, *OsABCC10*, and *OsABCG11*) were significantly upregulated (Table 2).

### 2.7. GST Activity

**Figure 9:**
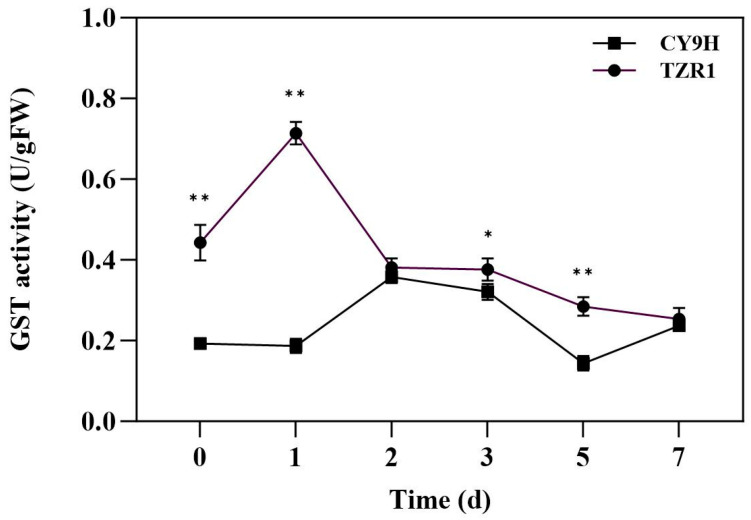
GST activity in CY9H and TZR1 at different days after topramezone treatment (*n* = 3). Topramezone was applied at 45 g a.i. ha^−1^. Data are expressed as the mean ± standard error (SE) from three biological replicates. Asterisks indicate significant differences of the GST activity in CY9H and TZR1 (* *p* < 0.05, ** *p* < 0.01).

The results of the GST activity indicated that compared to before the topramezone treatment, the GST activity of CY9H was decreased by 3.21% at 1 d, increased, respectively, by 85.49% and 66.32% at 2 and 3 d, decreased by 25.39% at 5 d, and increased by 22.80% at 7 d. The GST activity of TZR1 was increased by 61.17% at 1 d and decreased by 14.00~42.66% at 2~7 d (Figure 9). These results indicate that the GST activity fluctuated during the 7-day period after topramezone treatment. In addition, the GST activity of TZR1 was higher than that of CY9H at all timepoints, particularly at 0, 1, 3 and 5 d, where it significantly increased to 2.30-, 3.82-, 1.17-, and 1.98-fold, respectively (*p* < 0.05 or *p* < 0.01, *t*-test). The results showed that topramezone treatment had a time-course effect on the induction of GST activity. At 1 d, the GST activity in TZR1 was increased to the highest level and was significantly higher than that in CY9H, indicating that GST may play an important role in the metabolism of topramezone at this time.

### 2.8. Activity of Redox-Related Enzymes H_2_O_2_

**Figure 10:**
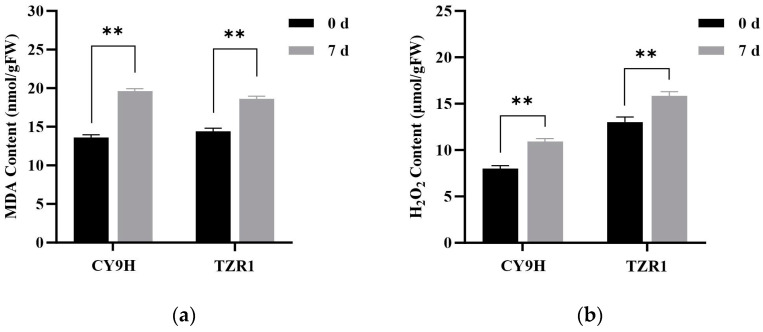

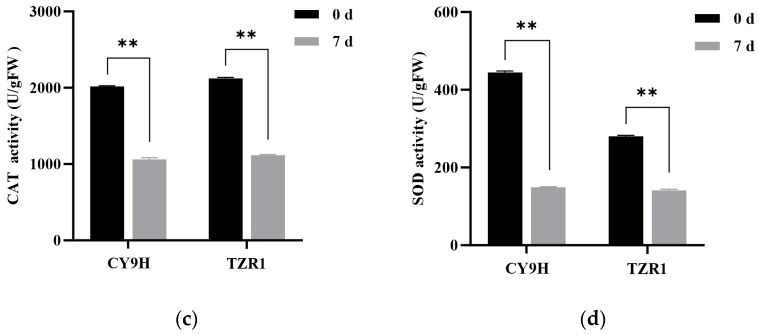
Redox-related enzyme activities in CY9H and TZR1 at 0 and 7 d after topramezone treatment: (**a**) MDA content; (**b**) H_2_O_2_ content; (**c**) CAT enzyme activity; and (**d**) SOD enzyme activity (*n* = 3). Topramezone was applied at 45 g a.i. ha^−1^. Data are expressed as the mean ± standard error (SE) from three biological replicates. Asterisks indicate significant differences in redox-related enzyme activities in CY9H and TZR1 (** *p* < 0.01).

Compared to the untreated controls, the malondialdehyde (MDA) content in TZR1 and CY9H was increased by 22.64% and 47.54% at 7 d, respectively (Figure 10a), while hydrogen peroxide (H_2_O_2_) content was decreased by 22.64% and 44.32%, respectively (Figure 10b). The catalase (CAT) activity in TZR1 and CY9H was decreased by 47.54% and 47.41%, respectively (Figure 10c), and superoxide dismutase (SOD) activity was decreased by 49.66% and 66.52%, respectively (Figure 10d). These results indicate that after topramezone treatment, the increase in MDA and H_2_O_2_ contents in TZR1 was lower than that in CY9H, and the decrease in SOD activity in TZR1 was also lower than that in CY9H, suggesting that TZR1 possesses stronger antioxidant capacity than CY9H.

## 3. Discussion

As one of the most widespread applications and extensions of simplified and labor-saving cultivation technologies in rice production, weed management, mainly depending on the use of chemical herbicides, has encountered significant challenges. In modern agricultural systems, the failure of weed control can result in over a 40% reduction in rice yield [23]. However, the prolonged use of single-class herbicides with the same mode of action (MoA) often leads to the development of different resistance levels in weeds. To date, 273 weed species, including 117 monocots and 156 dicots, have evolved to have resistance to herbicides [19,26]. The issue of herbicide-resistant weeds is becoming increasingly prominent in paddy fields. Most conventional herbicides are ineffective for weedy rice, which is both taxonomically and physiologically similar to cultivated rice. In other words, no chemical compounds have been identified that effectively control weedy rice without harming cultivated rice [23,27]. Therefore, the development of herbicide-resistant rice will provide significant potential for managing weeds and weedy rice resistant to herbicides with the same MoA. The scientific and reasonable application of multiple herbicide-resistant rice varieties in rotation could effectively mitigate the evolution of resistant weed populations.

Mutagenesis breeding involves inducing heritable changes in the DNA of rice through chemical or physical methods [28]. Compared to spontaneous mutations, mutagenesis breeding offers a shorter timeline and can enrich the gene pool for genetic improvement [29]. Developing herbicide-resistant rice through mutagenesis breeding is a feasible and practical strategy. In this study, the topramezone-resistant rice mutant TZR1, was selected from CY9H with the combination of radiation-induced mutagenesis and topramezone selection. Compared to CY9H, the RI value of TZR1 was increased to 1.94-fold. If base mutations or deletions occur in the TSR, topramezone may be hindered in its binding to OsHPPD enzymes, leading to reduced herbicidal efficacy. Sequence analysis of the full-length CDS of the *OsHPPD* gene in TZR1 did not reveal any base mutations or deletions. Consequently, topramezone can effectively interact with the OsHPPD enzyme, resulting in the expected herbicidal damage. These findings are consistent with the majority of published studies indicating that no mutations or deletions of the target sites are present in herbicide-resistant plants [30,31,32]. Furthermore, no significant differences in the expression levels of the *OsHPPD* mRNA were observed in TZR1 and CY9H at 0 and 12 h after topramezone treatment. Thus, these results suggest that the resistance of TZR1 to topramezone does not involve the TSR of HPPD.

Plants have evolved complex detoxification systems to fight against ever-changing environmental stressors. The NTSR is a key defense strategy in plants against toxic chemicals and involves a multi-phase detoxification process, including activation, conjugation, sequestration, and degradation. Genes related to metabolic detoxification, including those in the CYP450, GST, UGT and ABC transporter families, are integral to this process [33,34]. In this study, the experimental results of the detoxification enzyme inhibitors revealed that the CYP450 inhibitor malathion could not change the resistance of TZR1 to topramezone, whereas the GST inhibitor NBD-Cl effectively reversed it. Additionally, the GST activity in TZR1 was consistently higher than that in CY9H, particularly at 1 d after topramezone treatment, where a significant increase was observed. These results suggest that GST may mediate the resistance of TZR1 to topramezone.

GSTs in plants represent a multifunctional gene family, playing a crucial role in the metabolism and detoxification of herbicides. GSTs are considered to be key components involved in the second phase of herbicide detoxification. The diversity and dynamic evolution of the GST gene family enable them to participate in the detoxification processes of various toxic chemicals in plants [34]. Several GST genes have been identified as being associated with herbicide resistance in plants [35,36,37,38,39,40]. For example, the overexpression of *OsGSTL1* and *OsGSTL2* enhanced rice resistance to chlorsulfuron and glyphosate [35,36]. Additionally, the *ZmGST34* gene in maize heterologously expressed in *Escherichia coli* (Migula) Castellani and Chalmers could metabolize chloroacetylphenylamine herbicides, while the *ZmGST34* transgenic expressed in *Arabidopsis thaliana* (L.) Heynh showed resistance to those herbicides [37]. In soybean, overexpression of the *GmGSTU4* gene via genetic engineering in tobacco enhanced resistance to alachlor [38]. In weeds, for instance, the overexpression of *PfGSTF2* resulted in increased enzyme production, and it detoxified quizalofop-p-ethyl by conjugating with glutathione (GSH), thereby reducing the herbicide content. Moreover, the overexpression of *PfGSTF2* also scavenged reactive oxygen species (ROS) to minimize cellular damage and conferred the resistance of *Polypogon fugax* Nees ex Steud to quizalofop-p-ethyl [39]. Similarly, the overexpression of the *EcGSTU23* gene in *Echinochloa crus-galli* (L.) Beauv. conferred the resistance of transgenic maize to metamifop. Purified *EcGSTU23* catalyzed the rapid metabolism of metamifop when conjugated with GSH [40]. In the present study, the RNA-Seq analysis revealed that before and after topramezone treatment, DEGs were enriched in the Glutathione metabolism pathway in the KEGG analysis. Glutathione metabolism is linked to detoxification and GSTs are known to conjugate with GSH for herbicide detoxification. The verification results of the RT-qPCR assay indicate that five genes, including *OsGST4*, *OsGSTF1*, *OsGSTFU6-1*, *OsGSTU6-3*, and *OsGSTT3*, were significantly upregulated in TZR1 12 h after topramezone treatment, compared to the CY9H, suggesting that these genes may play a crucial role in the development of the resistance of TZR1 to topramezone.

UGTs play a crucial role in detoxifying various toxic chemicals, including herbicides, through glycosylation. Specifically, UGTs catalyze the conjugation of herbicides with glucuronic acid, resulting in more water-soluble and less toxic compounds, thereby facilitating their elimination from the organism [41]. For example, the overexpression of *AtUGT91C1* enhanced the resistance of *A. thaliana* to sulcotrione, while the *AtUGT91C1* mutant showed severe damage from this herbicide [42]. In apple (*Malus domestica* Borkh.), *MdUGT73CG22* metabolized and detoxified nicosulfuron and *MdUGT91AJ2* detoxified sulcotrione [43,44]. *LbUGT72B10* in *Lycium barbarum* (L.) is capable of converting 3,4-dichloroaniline, a herbicide degradation product, into its N-glucoside form. This conversion reduced the toxicity of 3,4-dichloroaniline and mitigates environmental pollution [45]. In *Aegilops tauschii* Coss., the upregulated expression of *UGT73C* and *UGT13248* may contribute to plant resistance to mesosulfuron-methyl [46]. ABC transporters are one of the largest protein families in plants and are conserved across all developmental stages of life. These transporters play a critical role in the detoxification process, particularly in phase III, by transporting herbicide molecules into vacuoles or out of cells [34,47]. The overexpressed *OsABC* (LOC_Os05g04600) facilitates the metabolism and transformation of bentazone, leading to the increased accumulation of endogenous metabolites, thereby reducing both the toxicity and residue levels of bentazone in rice. These results suggest that the overexpression of *OsABC* enhances rice plant resistance to bentazone [48]. Additionally, the overexpression of *OsABCG52* increased chlorophyll content, improved growth phenotypes, reduced ametryn accumulation, promoted its transport for catabolism, and conferred the resistance of rice to ametryn [49]. The *EcABCC8* in *E. crus-galli* can export glyphosate from the cytoplasm, decreasing intracellular glyphosate levels and mitigating its toxic effects [50]. *EcABCB4*, *EcABCB1*, and *EcABCB19* regulated auxin accumulation and mediated the resistance of *E. crus-galli* to dichlorprop [51]. Additionally, the expression of *AmABCC1* or *AmABCC2* from *Alopecurus myosuroides* Huds. enhanced the resistance of transgenic yeast to mesosulfuron-methyl [52]. These findings suggest that both UGTs and ABC transporters can contribute to the resistance of herbicides in plants; their interaction may further enhance the capacity of plants to detoxify and resist herbicide damage.

Researchers have found that not only the overexpression of individual resistance genes but also the co-regulation of multiple detoxification genes can enhance the resistance of plants to herbicides [33]. In the common wild oat (*Avena fatua* L.), both CYP450 and GST enzymes are involved in the detoxification process of clodinafop-propargyl [53], and the use of safeners has been shown to induce the upregulation of CYP450s, GSTs, UGTs, and transporter genes in maize [54]. In this study, 12 h after topramezone treatment, the results of GO enrichment analysis revealed that DEGs were enriched in the UDP-galactosyltransferase activity and transport pathways. The verification results of the RT-qPCR assay showed that *OsUGT76D1-1*, *OsUGT75L6*, *OsABCC8*, *OsABCC10*, and *OsABCG11* were significantly upregulated, suggesting that these genes may together be involved in the resistance of TZR1 to topramezone.

The application of herbicides induces abiotic stress, leading to the accumulation of ROS in plants, which can cause significant damage to them. However, antioxidant enzymes play a crucial role in scavenging ROS, thereby reducing oxidative damage to plants [55]. For instance, the *Y-EPSPS* gene of maize exhibit enhanced the resistance of transgenic *A. thaliana* plants to glyphosate; after glyphosate treatment, the activities of peroxidase, SOD, and CAT were significantly increased compared to those in wild-type plants [56]. Our previous research also demonstrated that rice plants treated with pyrroloquinoline quinone increased the activities of the above three enzymes, thereby enhancing the antioxidant capacity of rice and reducing herbicide-induced damage [57]. In the present study, 7 d after topramezone treatment, the increased levels of the MDA and H_2_O_2_ in TZR1, as well as the decreased activity of SOD, were less than those or that in CY9H, respectively. These results indicate that TZR1 was subjected to less oxidative stress than CY9H and consequently suffered less herbicide-induced damage. These results suggest that TZR1 may possess enhanced antioxidant defense mechanisms, which contribute to its greater resistance to oxidative stress and reduced injury from the HPPD inhibitor herbicides.

## 4. Materials and Methods

### 4.1. Rice Line, Herbicide, and Chemicals

The rice line CY9H was developed from indica rice cultivar Chuangyu 9, Hunan Rice Research Institute and Changsha Dahe Technology Development Center. Topramezone 4% oil dispersion, was supplied by Hunan Xinchangshan Agricultural Development Co., Ltd., Changde, China. The GST inhibitor, 4-chloro-7-nitrobenzoxadiazole (NBD-Cl, 98%), was purchased from Macklin Biochemical Technology Co., Ltd., Shanghai, China. The CYP450 inhibitor, malathion 45% emulsifiable concentrate, was purchased from Ningbo Sunjoy AgroScience Co., Ltd., Ningbo, China.

### 4.2. Plant Materials and Growth Conditions

Seeds of CY9H were treated with 180 Gy heavy ion beams to construct an M2 generation rice population. Thirteen surviving plants were screened from the M2 generation with topramezone at 150 g a.i. ha^−1^; their seeds were collected to construct an M3 generation rice population. The resistant mutant TZR1 was subsequently screened from the M3 generation with topramezone at 90 g a.i. ha^−1^. Additionally, M2 generation seeds were propagated in Sanya City, Hainan Province, China to obtain M3 generation seeds. For the experiments, M3 generation seeds of TZR1 and CY9H seeds were surface-sterilized by soaking in a 0.13% sodium hypochlorite solution for 12 h, followed by germination in a growth chamber (RDN-1000E-4, Ningbo Dongnan Instrument Co., Ltd., Ningbo, China). When rice seedlings reached approximately 5 cm in height, they were transplanted into small pots containing nutrient soil (1:1 *v*/*v* a mixture of rice substrate and vermiculite). Transplanted plants were cultivated in a growth chamber under the following controlled conditions: temperature 30 ± 2 °C; relative humidity 70–80%; a 14 h light period at 30,000 lux intensity, and a 10 h dark period at 0 lux intensity. These conditions were maintained consistently in subsequent experiments.

### 4.3. Topramezone Dose-Response Assay

CY9H and TZR1 plants were grown in a growth chamber until they reached the 3–4 leaf stage, after which a whole-plant dose–response assay was performed. Seedlings were picked up to retain six uniformly sized plants per pot. Topramezone was applied at 0, 15, 30, 45, 60, and 90 g a.i. ha^−1^ using a 3WP-2000 walking spray tower (Nanjing Research Institute for Agricultural Mechanization, Ministry of Agriculture and Rural Affairs, Nanjing, China). Each dose was replicated three times for each rice line. At 21 d after treatment, above-ground biomass was harvested, and the fresh weight was determined. The GR_50_ values of topramezone in the two rice lines were calculated based on the results of fresh weight. Then, the RI value was determined as the ratio of the GR_50_ value of topramezone in the TZR1 rice line divided by the GR_50_ value of topramezone in the CY9H rice line.

### 4.4. Sequencing of the OsHPPD Gene

At the 3–4 leaf stage, CY9H and TZR1 rice leaves were collected and snap-frozen in liquid nitrogen. Total RNA was extracted using the Trizol Total RNA Extraction Kit (GenStone Biotech, Yuyao, China) based on the protocol of the manufacturer. For sequencing of the *OsHPPD* gene, total RNA was reverse-transcribed into cDNA using the All-in-One 5× RT MasterMix (Applied Biological Materials Inc., Richmond, BC, Canada). Primers for amplification were designed using Primer Premier 5 (PREMIER Biosoft International, Palo Alto, CA, USA) (Table S1) based on the sequence of the referencce gene (*Os02g0168100*) retrieved from the China Rice Data Center (https://www.ricedata.cn/gene/. accessed on 6 May 2024). PCR was performed in a 25 μL reaction mixture containing 1 μL cDNA as the template; 0.5 μL KOD FX DNA polymerase (TOYOBO (Shanghai) Biotech Co., Ltd., Shanghai, China); 12.5 μL 2× PCR Buffer; 5 μL 2 mM dNTPs; 1 μL each of the primers; and 4 μL ddH_2_O. The PCR procedure was as follows: 94 °C for 2 min; 35 cycles of 98 °C for 10 s; 56 °C for 30 s; 68 °C for 1 min 20 s; and 68 °C for 5 min. PCR products were separated by agarose gel electrophoresis and target bands were excised and purified by a Gel Extraction Kit (Omega Bio-tek, Norcross, GA, USA). Purified products were sequenced by Beijing Tsingke Biotech Co., Ltd. (Beijing, China); sequence analysis was performed by SnapGene 7.0.2 software.

### 4.5. Determination of OsHPPD Gene Expression

At the 3–4 leaf stage, the CY9H and TZR1 plants were treated with topramezone at 5 g a.i. ha^−1^. Total RNA was extracted from the leaves of both treated and untreated plants at 12 h post treatment. Reverse transcription of total RNA into cDNA was performed by the StarScript Pro All-in-One RT Mix with gDNA Remover cDNA synthesis kit (GenStar Biotechnology Co., Ltd., Beijing, China). The cDNA was stored at −20 °C. The primers of the *OsHPPD* gene for RT-qPCR assay were designed using Primer Premier 5 (PREMIER Biosoft International, Palo Alto, CA, USA). The primer design specifications included: a length of 18–24 bp; a melting temperature (Tm) of 55–60 °C; a GC content of 40–60%; and a PCR product length of 80–200 bp. Prior to the formal experiment, primer validation was conducted. The selected primers exhibited a normal amplification curve, a single-peaked dissociation curve, no primer dimers, and a cycle threshold (Ct) value within the range of 15–35 (Table S1). The RT-qPCR assay was conducted using β-actin as the internal reference gene with a reaction volume of 10 μL including: 5 μL PerfectStart Green qPCR SuperMix (TransGen Biotech Co., Ltd.,Beijing, China.); 2.5 μL 10-fold cDNA dilution as a template; and 1.25 μL primer pairs (0.5 μmol/L). The thermal cycling procedure was as follows: 94 °C for 30 s, followed by 40 cycles of 94 °C for 5 s and 60 °C for 30 s on the Applied Biosystems QuantStudio 3 system (Thermo Fisher Scientific Inc., Pittsburgh, PA, USA). Relative expression levels of the OsHPPD mRNA were analyzed based on the 2^−ΔΔCt^ method [58]. Each treatment was conducted in triplicate, with each condition including two technical replicates.

### 4.6. Effects of CYP450 and GST Inhibitors on Topramezone Resistance

CY9H and TZR1 plants were cultivated in a growth chamber until the 3–4 leaf stage. For the CYP450 inhibition assay, plants were sprayed with malathion, at 1000 g a.i. ha^−1^, 2 h before the topramezone treatment. For the GST inhibition assay, plants were sprayed with NBD-Cl at 270 g a.i. ha^−1^, 24 h before the topramezone treatment. Topramezone was applied at 0, 15, 30, 45, 60, and 90 g a.i. ha^−1^. Each treatment was performed in triplicate for both rice lines. At 21 d after the topramezone treatment, above-ground biomass was harvested, and the fresh weight was measured. The GR_50_ and RI values were calculated to evaluate the inhibitory effects of malathion and NBD-Cl on topramezone resistance.

### 4.7. Whole-Genome Resequencing of CY9H and TZR1

CY9H and TZR1 plants were grown to the 3–4 leaf stage, and three pots of each rice line were considered. Additionally, TZR1 was treated with topramezone at 45 g a.i. ha^−1^ to screen out plants with stable traits of topramezone resistance. Fourteen days post treatment, 100 mg of leaves from the TZR1 plants without phytotoxicity from topramezone and CY9H plants, were collected. Three repeats were conducted for each rice line. Then, leaf samples were snap-frozen in liquid nitrogen and stored at −80 °C until the WGS performed by Beijing Tsingke Biotech Co., Ltd., Beijing, China. Genomic DNA was extracted and assessed for quality. High-quality DNA was fragmented using ultrasonic shearing and subsequently purified. Fragmented DNA was subjected to end repair, 3′-end adenylation, and adapter ligation. Size selection of the fragments was performed by agarose gel electrophoresis, followed by PCR amplification to construct sequencing libraries. Libraries passing quality control were sequenced on the DNBSEQ-T7 platform (MGI Tech Co., Ltd., Shenzhen, China). Raw paired-end reads were assessed for quality; clean reads were obtained by filtering low-quality sequences. Clean reads were aligned to the reference genome of indica rice R498 (https://www.mbkbase.org/R498/. accessed on 9 August 2024). SNPs and Indels were identified and annotated based on the alignment results. Comparative genomic analysis was performed between CY9H and TZR1, focusing on the detected SNPs and Indels. Functional annotation and enrichment analyses of these variations were conducted using GO and KEGG pathway analysis. The top 20 pathways with the lowest *p*-values, ranked by the Gene Ratio, were identified.

### 4.8. Transcriptome Sequencing

When CY9H and TZR1 plants were cultivated at the 3–4 leaf stage, 100 mg leaf samples were collected from these plants at 0 and 12 h after treatment with topramezone at 5 g a.i. ha^−1^. For each sampling timepoint, leaf samples per rice line were conducted with three biological replicates. Leaf samples were snap-frozen in liquid nitrogen and stored at −80 °C until the RNA-seq. The total RNA was extracted, and its purity and integrity were assessed using the Agilent 2100 Bioanalyzer (Agilent Technologies, Inc., Santa Clara, CA, USA) and by 1.0% agarose gel electrophoresis. The RNA concentration was measured using the Qubit Fluorometer (Thermo Fisher Scientific, Waltham, MA, USA). RNA samples meeting the quality requirements for RNA-seq were used to construct cDNA libraries. RNA-seq was performed by Beijing Tsingke Biotech Co., Ltd., and conducted on the Illumina NovaSeq 6000 platform (Illumina, Inc., San Diego, CA, USA) using paired-end sequencing, ensuring that the Q30 base percentage exceeded 85%. Raw data obtained from the RNA-seq were filtered to generate clean reads. Clean reads were aligned to the reference genome of indica rice R498 using Hisat2, for obtaining positional information on the reference genome or genes and unique sequence features of the samples. Transcripts were assembled using StringTie, and expression levels were evaluated by the Fragments Per Kilobase of transcript per Million fragments mapped (FPKM) metric. Differential expression analysis between sample groups was conducted using DESeq2. DEGs were identified with thresholds of false discovery rate (FDR) < 0.05 and fold change (FC) > 2. Functional annotation and enrichment analyses of DEGs were performed using GO and KEGG pathway analysis. The top 20 pathways with the lowest *p*-values, ranked by the Gene Ratio, were identified.

### 4.9. RT-qPCR Validation of Differentially Expressed Genes

Based on the RNA-seq results, upregulated candidate genes (*p* < 0.05, |log2FC| > 1.0) involved in herbicide detoxification metabolism including GSTs, ABC transporters, and UGTs were selected to be validated using RT-qPCR assay, according to the experimental procedure described in Section 4.5. Leaf samples from CY9H and TZR1 were collected at 12 h after treatment with topramezone at 5 g a.i. ha^−1^. Total RNA was extracted and reverse-transcribed into cDNA; the cDNA was stored at −20 °C. Primers for target genes were designed using Primer Premier 5 (PREMIER Biosoft International) (Table S1). The primer design specifications, primer validation, reaction conditions, and system for the RT-qPCR assay were consistent with those in Section 4.5. Each treatment was conducted in triplicate, with each condition including two technical replicates.

### 4.10. Measurement of Enzymatic Activity

CY9H and TZR1 plants were cultivated to the 3–4 leaf stage in a growth chamber and then treated with topramezone at 45 g a.i. ha^−1^. For the measurement of GST activity, 100 mg leaves per rice line were collected at 0, 1, 2, 3, 5, and 7 d after treatment, snap-frozen in liquid nitrogen, and stored at −80 °C until use. For each sampling timepoint, three biological replicates per rice line were considered. In addition, for the determination of MDA and H_2_O_2_ contents, as well as CAT and SOD activities, 100 mg leaves per rice line were collected at 0 and 7 d post treatment. Twelve biological replicates per rice line were collected for each sampling timepoint, snap-frozen in liquid nitrogen, and stored at −80 °C. The aforementioned enzymatic activities were measured using commercial assay kits (Beijing Solarbio Science & Technology Co., Ltd., Beijing, China) according to the manufacturer’s protocols, with the SpectraMax 190 (Molecular Devices, LLC., San Jose, CA, USA).

### 4.11. Data Analysis

The chemical structure of topramezone was drawn by ChemDraw 19.0 software (PerkinElmer Inc., Shelton, CT, USA). The GR_50_, with corresponding 95% CL, slopes of regression lines, and both chi-squared and *p*-values were calculated using a probit regression analysis with IBM^®^ SPSS^®^ Statistics Version 23 software (SPSS Inc., Chicago, IL, USA). The GR_50_ values were considered as significantly different when not overlapping with each other in the corresponding 95% CI (*p* < 0.05). The results from the RT-qPCR and enzymatic activity assays were analyzed using IBM^®^ SPSS^®^ Statistics, Version 23 software with *t*-tests. Statistically significant difference was indicated as *p* < 0.05 (*) or *p* < 0.01 (**). Graphs for *OsHPPD* gene expression levels and enzymatic activity data were generated using GraphPad Prism 10 (GraphPad Prism 10 Software, San Diego, CA, USA).

## 5. Conclusions

The topramezone-resistant rice mutant TZR1 was developed by a combination of irradiation mutagenesis and topramezone selection from CY9H. The *OsHPPD* gene and CYP450s were not responsible for the resistance of TZR1 to topramezone; however, GSTs were possibly responsible for topramezone resistance in the TZR1 line. Five GST genes (*OsGST4*, *OsGSTF1*, *OsGSTFU6-1*, *OsGSTU6-3*, and *OsGSTT3*), two UGT genes (*OsUGT76D1-1* and *OsUGT75L6*), and three ABC transporter genes (*OsABCC8*, *OsABCC10*, and *OsABCG11*) were significantly upregulated in TZR1, suggesting that they may be involved in the resistance of TZR1 to topramezone. Additionally, other redox-related enzymes, including MDA, H_2_O_2_, and SOD were possibly associated with the increased antioxidant capacity of TZR1. These findings suggested that GSTs may play a main role in the resistance of TZR1 to the HPPD inhibitor topramezone. The results will provide a scientific reference for the application and extension of topramezone-resistant rice for managing weeds and weedy rice resistant to herbicides with the same MoA in paddy fields.

## Figures and Tables

**Table 1:** Effects of malathion and NBD-Cl on the resistance of CY9H and TZR1 to topramezone.

Rice Line	Treatment	Slope ± SE	Chi-Square (X^2^)	df	*p*-Value	GR50 (95%CL) (g a.i. ha^−1)^^#^	RI ^§^
CY9H	Topramezone	3.761 ± 0.414	2.433	3	0.487	31.27 (27.10–35.36) ^b^	1.00
	Topramezone + Malathion	3.644 ± 0.412	1.800	3	0.615	37.81 (33.15–42.76) ^b^	1.21
	Topramezone + NBD-Cl	3.828 ± 0.419	2.081	3	0.556	35.55 (31.21–40.02) ^b^	1.14
TZR1	Topramezone	3.440 ± 0.436	0.156	3	0.984	60.51 (52.93–71.51) ^a^	1.94
	Topramezone + Malathion	3.418 ± 0.437	0.220	3	0.974	62.19 (54.27–73.94) ^a^	1.99
	Topramezone + NBD-Cl	3.321 ± 0.398	2.287	3	0.515	36.00 (31.09–41.12) ^b^	1.15

^#^ GR_50_ indicates the dose of tested chemical(s) for a growth reduction by 50% in fresh weight (*n* = 108). The GR_50_ values of each treatment followed by different lower-case letters in the same column indicate a significant difference when not overlapping with each other in the corresponding 95% confidence limits (CL) (*p* < 0.05). ^§^ RI denotes resistance index: resistance index = GR_50_ value of tested chemical(s)/GR_50_ value of topramezone in the CY9H rice line.

**Table 2:** Verification of upregulated DEGs in CY9H and TZR1 by RT-qPCR assay.

DEGs	Function Annotation	Log_2_FC ^a^	FC ^b^	*t*-Test ^c^
GSTs	*OsGST4*	1.48	2.74	**
	*OsGSTF1*	5.37	6.75	**
	*OsGSTU6-1*	1.62	2.06	*
	*OsGSTU6-3*	13.40	7.35	**
	*OsGSTT3*	9.29	2.40	*
UGTs	*OsUGT73D1*	2.90	2.93	*
	*OsUGT75L6*	1.79	3.71	*
ABC transporter	*OsABCC8*	1.33	3.50	*
	*OsABCC10*	3.72	20.44	**
	*OsABCG11*	2.32	19.76	**

^a^ RNA-seq results (*n* = 3). FC, fold change. ^b^ RT-qPCR results (*n* = 6). ^c^ Asterisks indicate significant differences of DEGs in CY9H and TZR1 (* *p* < 0.05, ** *p* < 0.01).

## Data Availability

The datasets generated for this study can be found in the online repositories. The names of the repository/repositories and accession number(s) can be found below: https://www.ncbi.nlm.nih.gov/ (accessed on 19 December 2024), PRJNA1197743 and PRJNA1198727.

## Supplementary Materials

The following supporting information can be downloaded at: https://www.mdpi.com/article/10.3390/plants14030425/s1, Table S1: Primers used in this study.

## Abbreviations

The following abbreviations are used in this manuscript:

CY9H	Chuangyu 9H
HPPD	4-hydroxyphenylpyruvate dioxygenase
TSR	Target-site resistance
NTSR	Non-target-site resistance
CYP450s	Cytochrome P450 monooxygenases
GSTs	Glutathione S-transferases
UGTs	UDP-glycosyltransferases
ABC	ATP-binding cassett
EMS	Ethyl methanesulfonate
RT-qPCR	Real-time quantitative PCR
WGS	Whole-genome resequencing
DEGs	Differentially expressed genes
RNA-seq	Transcriptome sequencing
GR_50_	The herbicide dose for a growth reduction by 50%
RI	Resistance index
NBD-Cl	4-chloro-7-nitrobenzoxadiazole
GO	Gene Ontology
DGs	Differential genes
KEGG	Kyoto Encyclopedia of Genes and Genomes
MDA	Malondialdehyde
H_2_O_2_	Hydrogen peroxide
CAT	Catalase
SOD	Superoxide dismutase
MoA	Mode of action
GSH	Glutathione
ROS	Reactive oxygen species
FC	Fold change
FPKM	Fragments Per Kilobase of transcript per Million fragments mapped
FDR	False discovery rate
CL	Confidence limits
